# Identification of unique expression signatures and therapeutic targets in esophageal squamous cell carcinoma

**DOI:** 10.1186/1756-0500-5-73

**Published:** 2012-01-26

**Authors:** Wusheng Yan, Joanna H Shih, Jaime Rodriguez-Canales, Michael A Tangrea, Kris Ylaya, Jason Hipp, Audrey Player, Nan Hu, Alisa M Goldstein, Philip R Taylor, Michael R Emmert-Buck, Heidi S Erickson

**Affiliations:** 1 Pathogenetics Unit, Laboratory of Pathology, National Cancer Institute, Bethesda, USA; 2 Biometric Research Branch, Division of Cancer Treatment and Diagnosis, National Cancer Institute, Bethesda, USA; 3 Tissue Array Research Program, Laboratory of Pathology, National Cancer Institute, Bethesda, USA; 4 Laboratory of Pathology, National Cancer Institute, Bethesda, USA; 5 Microarray Facility, National Cancer Institute, Bethesda, USA; 6 Genetic Epidemiology Branch, Division of Cancer Epidemiology and Genetics, National Cancer Institute, Bethesda, USA; 7 Department of Thoracic/Head and Neck Medical Oncology, UT MD Anderson Cancer Center, Houston, USA

## Abstract

**Background:**

Esophageal squamous cell carcinoma (ESCC), the predominant histological subtype of esophageal cancer, is characterized by high mortality. Previous work identified important mRNA expression differences between normal and tumor cells; however, to date there are limited *ex vivo *studies examining expression changes occurring during normal esophageal squamous cell differentiation versus those associated with tumorigenesis. In this study, we used a unique tissue microdissection strategy and microarrays to measure gene expression profiles associated with cell differentiation versus tumorigenesis in twelve cases of patient-matched normal basal squamous epithelial cells (NB), normal differentiated squamous epithelium (ND), and squamous cell cancer. Class comparison and pathway analysis were used to compare NB versus tumor in a search for unique therapeutic targets.

**Results:**

As a first step towards this goal, gene expression profiles and pathways were evaluated. Overall, ND expression patterns were markedly different from NB and tumor; whereas, tumor and NB were more closely related. Tumor showed a general decrease in differentially expressed genes relative to NB as opposed to ND that exhibited the opposite trend. FSH and IgG networks were most highly dysregulated in normal differentiation and tumorigenesis, respectively. DNA repair pathways were generally elevated in NB and tumor relative to ND indicating involvement in both normal and pathological growth. PDGF signaling pathway and 12 individual genes unique to the tumor/NB comparison were identified as therapeutic targets, and 10 associated ESCC gene-drug pairs were identified. We further examined the protein expression level and the distribution patterns of four genes: ODC1, POSTN, ASPA and IGF2BP3. Ultimately, three genes (ODC1, POSTN, ASPA) were verified to be dysregulated in the same pattern at both the mRNA and protein levels.

**Conclusions:**

These data reveal insight into genes and molecular pathways mediating ESCC development and provide information potentially useful in designing novel therapeutic interventions for this tumor type.

## Background

Esophageal cancer (EC) is the eighth most common cancer in the world and has the sixth highest mortality [1,2]. As the predominant histological subtype of esophageal cancer, esophageal squamous cell carcinoma (ESCC) comprises 80% of all esophageal cancer worldwide. Despite advances in diagnostic methods and combined treatment modalities, the majority of tumors are diagnosed at advanced stages and the overall 5-year survival rate (1999 to 2005) is only 19% [3]. In contrast, the 1% of patients who are diagnosed with Stage I disease (T1N0M0), invading only the lamina propria or submucosa without lymph node or distant metastasis, have a favorable 90% 5-year survival after resection [4]. Therefore, early diagnosis of ESCC is important in preventing this cancer, and there is a significant need to develop novel therapeutic agents for patients with advanced disease. Moreover, ESCC shares many phenotypic and molecular characteristics with both squamous cell carcinoma of the head and neck, and of the lung, both of which are significant clinical problems [5-7], thus new molecular insights or drug targets discovered by studying ESCC may have widespread utility for reducing cancer morbidity and mortality.

The normal esophagus is lined by a stratified squamous epithelium composed of three main layers: superficial, differentiated, and basal [8]. The epithelial basal cells are adjacent to the basement membrane, are cuboidal or polyhedral in shape, and comprise a layer that is one to four cells thick and accounts for 15% or less of the epithelium [9]. The basal layer contains proliferating stem cells and transit-amplifying cells that migrate towards the luminal surface during normal maturation of the esophageal epithelium [10,11]. However, in early squamous cell cancer formation an atypical proliferation of the basal cell compartment is observed, initially confined to the lower part of the epithelium then increasingly extending through the entire epithelium [12]. Over time the proliferating cells occupy the full thickness of the epithelium and ultimately invade through the lamina propria into the surrounding sub-epithelial tissue. Thus, a critical change that occurs during tumorigenesis is the inability of the basal cells to properly differentiate. To date, the histopathological characteristics of this cancer-related alteration have been described in detail, but the molecular pathways that mediate this change *in vivo *are less well known.

To contrast and compare the genes and molecular pathways that mediate these physiological and pathological states in patients, we microdissected normal squamous epithelial basal cells, normal squamous epithelial differentiated cells, and matched ESCC cells from twelve *ex vivo *clinical samples and evaluated expression profiles using microarrays. The primary aim of the study was to compare the growth-related genes in normal basal cells versus tumor cells in a search for new therapeutic targets, and hence, assess the feasibility of using a microdissection based strategy to identify novel therapeutic targets. As a first step towards this goal, we determined the global patterns of gene expression and specific pathways associated with cell differentiation versus those associated with tumorigenesis, and in parallel evaluated the expression patterns of DNA damage and repair related genes in ESCC since this tumor type exhibits significant genomic instability [13-16]. Uniquely, the current study uses microdissected normal basal epithelium as a 'growth filter' to identify genes and pathways specific to tumor growth.

## Methods

### Clinical tissue specimens

All cases and samples were obtained from subjects residing in the Taihang mountain region of north central China. This study was approved by the Institutional Review Boards of the collaborating institutions (Single Project Assurance Number# S-12118-01): Shanxi Cancer Hospital and Institute, Taiyuan, Shanxi Province, China; and the National Cancer Institute, Bethesda, MD, USA. After obtaining informed consent, cases were interviewed to obtain information on demographics, cancer risk factors (eg, smoking, alcohol drinking and detailed family history of cancer), and clinical information (Additional file 1: Table S3). None of the cases had prior therapy. Using accepted inclusion criteria [17], twelve cases having sufficient tumor and matched normal epithelium were evaluated and selected by a pathologist (J.R.-C.). Resected specimens from the 12 ESCC patients were fresh frozen, blocked and stored at LN2 according to standard practices [18] until assays could be performed. Four matched ethanol-fixed paraffin-embedded blocks from the above 12 cases, including both tumor and normal compartments were selected for protein validation by immunohistochemistry (IHC).

### Tissue processing

Immediately prior to use, the paired normal and tumor samples were cut into 8 μm thick sections using a Leica Cryostat, placed onto glass slides, and stored for less than two weeks at -80°C. Before dissection, each section was individually removed from storage and immediately stained and dehydrated using an H&E protocol designed for microdissection [19-21].

### Laser capture microdissection

**Figure 1:**
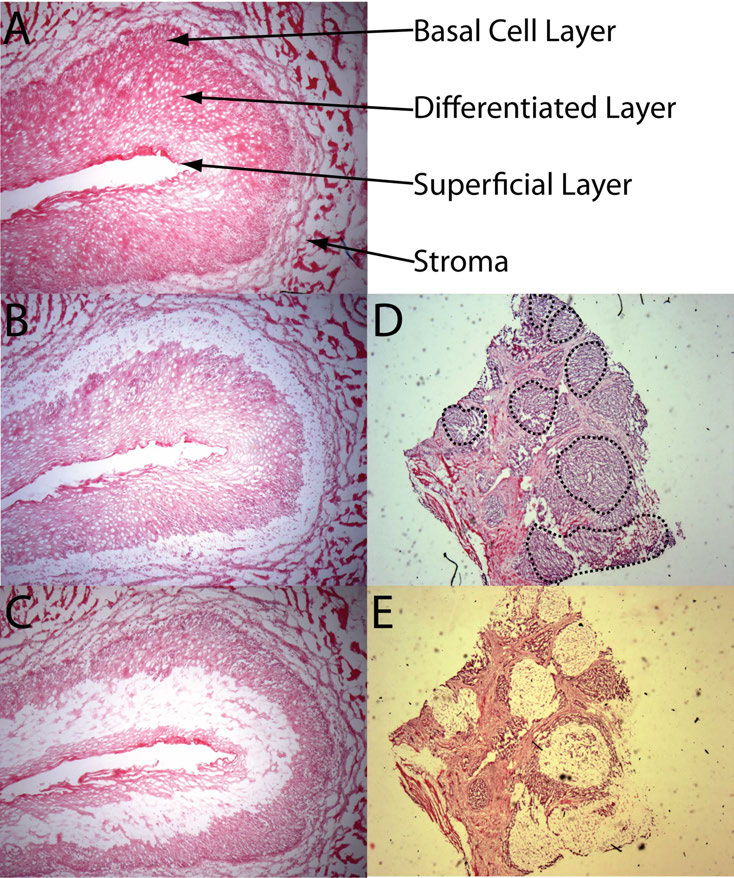
**Histology of normal esophageal epithelium and ESCC before and after microdissection**. **A**. Hematoxylin and eosin (H&E) staining showing the three layers of normal esophageal epithelium. **B**. Normal esophageal epithelium after basal layer microdissection. **C**. Normal esophageal epithelium after differentiated layer microdissection. **D**. ESCC clusters (Black circles) before microdissection. **E**. ESCC clusters (blank areas) after microdissection. Magnification: A = 10×, B = 10×, C = 10×, D = 4×, E = 4×

For each case, paired normal and tumor sections were chosen and morphologically selected cell populations were dissected by laser capture microdissection with the PixCell IIe (Arcturus Engineering, Inc., Mountain View, CA) [22], according to standard protocols [20]. After H&E staining, the normal basal layer of the squamous epithelium, consisting of 1-4 cells thick, including the strata basale and parabasale immediately adjacent to the basement membrane, was procured by LCM (Figure 1). Next, the differentiated region of the epithelium, consisting of stratum spinusm, stratum granulosum and stratum lucidum cell layers was identified and retrieved after LCM. Since the molecular profile comparison between the regions was critical in this study, overlapping of the two cellular subtypes was strictly avoided during microdissection. Corresponding ESCC was microdissected from matched tissue blocks in each case using the same LCM protocol.

### RNA isolation and assessment

The time from slide removal from the freezer to completion of LCM did not exceed 30 minutes. Following LCM, RNA was isolated using the PicoPure RNA Isolation kit (Arcturus Engineering, Inc. Mountain View, CA) digested with DNase, and the quantity and quality measured as previously described [19,21] using the Bioanalyzer 2100 (Agilent Technologies, Inc., Palo Alto, CA) and NanoDrop ND-1000 Spectrophotomer (NanoDrop Technologies, Wilmington, DE), respectively.

### RNA amplification, fragmentation, hybridization and microarray

Total RNA was used as the template for amplification because of bias reduction compared to using mRNA template [23]. To ensure that all samples contained a similar overall representation of transcriptome, 50 ng of total RNA concentrations were used for each sample. Two rounds of linear amplification were performed by combining reagents supplied in MessageAmp™ II-Biotin Enhanced Single Round aRNA Amplification Kit (Ambion, Catalog# AM1791, Austin, TX) and MessageAmp™ II aRNA Amplification Kit (Ambion, Catalog# AM1751), resulting in biotin-labeled antisense cRNA [24]. Per sample,15 μg of biotin-labeled cRNA sample was fragmented and processed for hybridization to Human Genome U133A 2.0 Genechips (Affymetrix Inc., Santa Clara, CA), according to the Expression Kit User Manual http://www.affymetrix.com/support/technical/manuals.affx). Arrays were washed and stained using the Midi_euk2v3_450 protocol (V4). Fluorescent intensity emitted by the labeled target was measured using a GeneChip Scanner 7 G (Affymetrix Inc.). The microarray gene expression data can be accessed via GEO repository through the following link: http://www.ncbi.nlm.nih.gov/geo/query/acc.cgi?acc=GSE29001

### Data analysis and statistics

#### (1) Microarray data quality control, preprocessing and gene Filtering

Quality assessment of each microarray was done using probe level model based quality statistics: normalized unscaled standard error (NUSE) and relative log expression (RLE). Arrays with their respective median NUSE and RLE values exceeding the upper control limits were considered as poor quality and excluded from analysis. Only one of the 36 arrays did not pass the quality control criteria (Additional file 1: Figure S1). Robust Multi-Array (RMA) with quantile normalization was completed for the 35 good quality arrays using the Bioconductor suite of array analysis tools running in R version 2.8.0 (R Development Core Team, 2004). Probe sets showing minimal variation across the 35 arrays were excluded from the analysis. Probe sets were selected if their expression differed by at least 1.5 fold from the median in at least 20% of the arrays. Overall, 10,725 probe sets were retained and used for analysis.

#### (2) Hierarchical clustering

Agglomerative hierarchical clustering was used to inspect the global grouping of expression profiles from tumors and normal tissues of the twelve patients. Both specimens and genes were clustered with a 1-correlation metric employing average linkage in R.

#### (3) Class comparison

Differentially expressed genes between subgroups of specimens were determined by multivariate permutation test [25]. To control for the proportion of false positives, the multivariate permutation test was used to provide a less than 5% false positive rate with 95% confidence. The test statistics used were the paired t-statistics for each probe set. Although paired t-statistics were used, the multivariate permutation test is non-parametric and does not require the assumption of normal distributions for gene expression measurements. The multivariate permutation test was performed using BRB-Array Tools version 3.8.0 software. Data of genes with > 2 fold change in the class comparison were then visualized by Principal Components Analysis (PCA). PCA maps were generated by input of sub-type cell populations related CEL files to Partek Genomics Suit (Partek, Inc., St. Charles, MO).

#### (4) Pathway analysis

Genes identified by class comparison (fold change > 2) as described above were used for network and gene ontology analyses. Data were analyzed through the use of Ingenuity Pathways Analysis (Ingenuity^® ^Systems, http://www.ingenuity.com). Gene accession numbers were imported into the Ingenuity Pathway Analysis system and networks of the focus genes were algorithmically generated based on their connectivity. Briefly, biochemical pathway analysis was performed by measuring the ratio of the number of molecules in a given pathway divided by the total number of molecules that make up that pathway to generate networks in which the differentially regulated genes are related to known associations between genes or proteins, but independent of established canonical pathways.

### Immunohistochemistry

Immunohistochemical (IHC) staining of ethanol-fixed and paraffin-embedded sections for protein expression of four genes of interest (ODC1: ornithine decarboxylase 1; POSTN: periostin; ASPA: aspartoacylase; and, IMP3: insulin-like growth factor 2 mRNA binding protein 3) was performed using a standard IHC protocol as we previously described [26]. IHC antibodies chosen represent available working antibodies in our laboratory, of which four represented proteins in which gene expression was dysregulated in T epithelium. Antibodies used included rabbit polyclonal anti-ODC1 primary antibody (Sigma) at 1:200, rabbit polyclonal anti-POSTN primary antibody (Sigma) at 1:300, rabbit polyclonal anti-ASPA primary antibody (Abcam) 1:300, and prediluted rabbit polyclonal anti-IMP3 primary antibody (Abcam). Negative controls were established by replacing the primary antibody with rabbit polyclonal IgG (Abcam).

## Results

### Technical parameters

A total of 36 microdissected samples from 12 patients were generated. Normal basal epithelial cells, normal differentiated epithelial cells, and tumor cells were procured from each patient specimen using 4000-8000 laser shots (Figure 1). Total RNA from 35 of the 36 samples was of sufficient quality for array analysis with a pre-amplification mean RNA Integrity Number (RIN) of 6.6 (range 4.2 to 8.7; Additional file 1: Table S1, Additional file 1: Figure S1).

### Profile and pathway analysis

We first determined the global patterns of gene expression and specific pathways associated with cell differentiation versus those associated with tumor growth and evaluated the expression patterns of DNA damage and repair related genes. This represents a cross-sectional study that is a good first step in generating a better molecular understanding of ESCC.

#### Differentiation versus tumorigenesis

**Figure 2:**
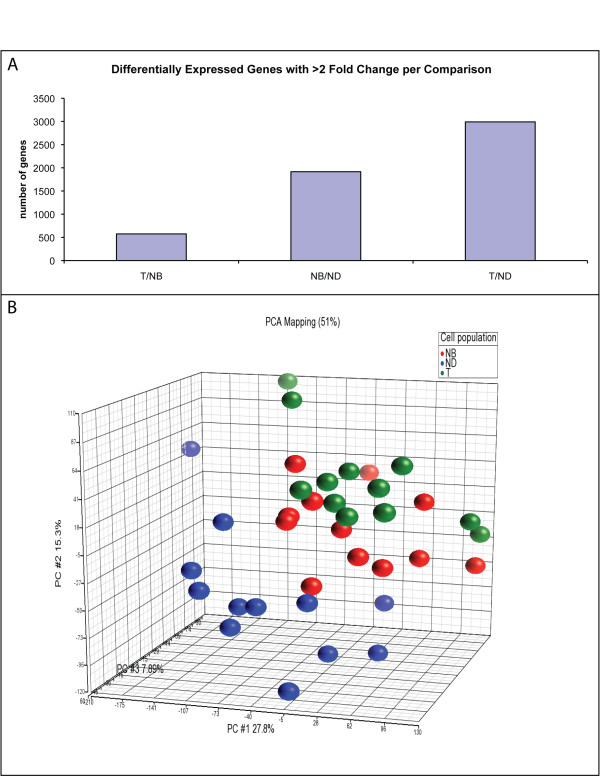
**Class comparison and principal components analysis (PCA) among the cell populations**. **A**. Class comparisons show the numbers of differentially expressed genes with > 2 fold change on each comparison. **B**. PCA mapping for the microarray data. The gene expression data (> 2 fold change) for each cell population are represented by different colors: normal basal cell (NB) gene expression = red; normal differential cell (ND) gene expression = blue; and tumor cell (T) gene expression = green.

**Figure 3:**
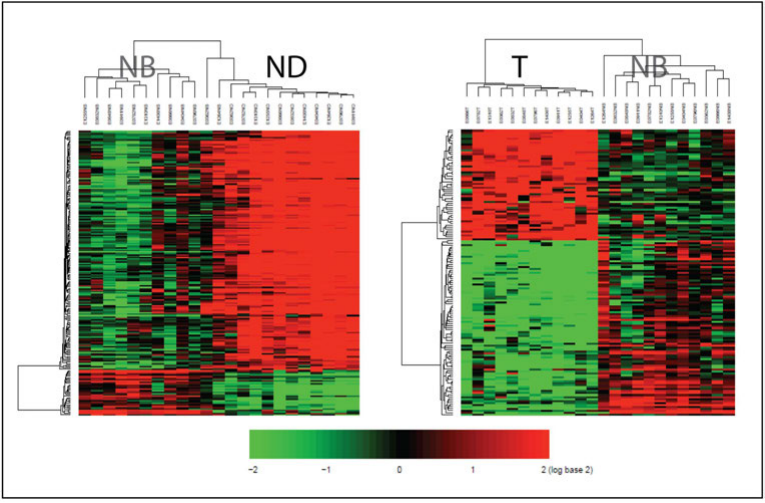
**Two-way hierarachical clustering on NB vs. ND and T vs. NB**. The differentially expressed genes with fold change > 4 in the NB vs. ND and T vs. NB comparisons were applied in this analysis. Each row represents a gene and each column corresponds to a sample. Expression of each gene was median-centered across all specimens for that gene. Shades of red represent degrees of increasing expression, and shades of green represent degrees of decreasing expression.

Initially, three sets of class comparisons were performed for the filtered probe sets: T vs. NB (T/NB); T vs. ND (T/ND); and NB vs. ND (NB/ND). Overall, the data showed that gene expression in normal differentiated cells was markedly different from both normal basal cells and tumor; whereas, the tumor and normal basal cells were more closely related. Specifically, for genes with a > 2 fold change, T/ND (2990) and NB/ND (1916) had a larger number of differentially expressed genes than T/NB which showed only 575 changes (Figure 2A). These data are reflected in the dendogram generated by non-supervised clustering shown in Figure 3. We also compared how the NB and ND cells clustered in comparison to tumor using PCA. Again, the ND cell samples were the most distinct based on location and distance from the NB and T cell populations, consistent with the number of differentially expressed genes (Figure 2B).

Since a large number of differentially expressed genes were initially identified among the four comparisons (above three comparisons plus T/N which represents T vs. NB + ND), the samples were secondarily filtered according to the highest geometric mean fold change to identify the set with the largest differences. A stringent level corresponding to at least 4-fold alteration was used to generate the top 10 up- and down-regulated genes for each comparison (Table 1). As expected, several of the top 10 up-regulated genes in ND (compared to T) were related to formation of the epidermis (e.g. TGM3 and SCEL) and were not altered in cancer. CRNN, also known as squamous epithelial heat shock protein 53, was highly up-regulated in ND (247.78 fold) and NB but not T, suggesting that the processes of calcium binding [27], mucosal/epithelial immune response and epidermal differentiation [28] are not altered in cancer. In addition, a comparison of expression profiles of stem-cell markers CD44 (↑ 4.369 fold in NB/ND and ↑ 2.918 fold in T/ND) and CD24 (↓ 3.789 fold in NB/ND and ↓ 14.275 fold in T/ND) revealed that the NB and T showed a stem cell-like high CD44 and low CD24 pattern relative to ND. The T/N comparison identified 286 genes dysregulated with at least a 4-fold change (Additional file 1: Figure S2). Using IPA, CRISP databases we identified a subset of 10 tumor dysregulated genes (AURKA↑, CDC2↑, PDGFRA↓, PDGFA↑, IL8RB↓, NPR3↓, NR3C2↓, ODC1↑, PRKCA↑, SOAT1↑) where therapeutic agents exist (MLN8054, flavopiridol, becaplermin, cilostazol, SB-265610, nesiritide, fludrocortisones acetate, eflornithine, L-threo-safingol, pactimibe; respectively) that act as inhibitors or agonists to the tumor dysregulated genes and are in clinical use or trial for other diseases. Moreover, twelve unique cancer-associated genes (IGF2BP3↑, HLF↓, SLC6A1↑, IL1R2↓, DNASE1L↓, ASPA↓, EDN3↓, PDCD4↓, COL14A1↓, RGS5↓, DEPDC6↓ and PAX5↓) were discovered using the dissected cell comparison of T/NB as they did not overlap with the genes identified in the tumor versus whole normal (NB + ND) comparison, demonstrating the technical value of the microdissection strategy employed in the study.

**Table 1:** Top 10 significant transcripts of each comparison

Comparison	Genes	Fold-change up-regulated	Genes	Fold-change down-regulated
T vs. N*	MMP1	17.234	CRNN	-127.008

	SPP1	16.244	CLCA4	-48.772

	LHX2	16.178	KRT13	-48.77

	MAGEA3	13.484	MAL	-47.356

	POSTN	12.907	SPRR3	-45.444

	CXCL11	12.892	TGM3	-43.083

	INHBA	12.846	KRT4	-36.124

	COL11A1	12.773	RHCG	-32.492

	KRT17	12.284	SPINK5	-25.795

	HOXA9	11.241	CYP4B1	-22.326

**T vs. NB**	KRT17	19.58	CRNN	-76.12

	SPP1	17.715	KRT13	-43.49

	MMP1	17.433	TFAP2B	-36.57

	LHX2	15.462	MAL	-34.49

	MAGEA3	14.996	KRT4	-34.27

	INHBA	13.668	SPRR3	-33.57

	COL11A1	13.558	CYP4B1	-31.25

	POSTN	10.788	CLCA4	-29.347

	HOXA9	9.405	RHCG	-22.791

	ISG15	9.148	SPINK5	-18.506

**T vs. ND**	MMP1	21.074	CRNN	-247.775

	CXCL11	20.169	TGM3	-130.782

	LHX2	18.786	MAL	-80.503

	POSTN	15.136	SCEL	-67.596

	MAGEA3	14.998	SPRR3	-64.667

	ECT2	14.384	SYNPO2L	-63.086

	TOP2A	14.229	KRT13	-58.246

	AURKA	13.738	SLURP1	-56.244

	SPP1	13.712	UPK1A	-55.326

	PTHLH	12.817	FLG	-52.983

**NB vs. ND**	TFAP2B	10.432	SYNPO2L	-20.626

	CXCL14	10.196	MXD1	-14.761

	SERPINE2	6.592	FLG	-13.662

	NCAPG	5.708	ANXA9	-12.746

	TOP2A	5.676	EREG	-12.346

	PTHLH	5.236	PRSS3	-12.131

	NDC80	5.223	DKK1	-11.945

	RRM2	5.199	ECM1	-11.893

	PPAT	4.928	MUC1	-11.814

	CH25H	4.561	ZNF365	-11.774

* N = NB + MD

We next compared and contrasted overall NB cell transcript levels versus that of ND and T cells to assess the directionality of gene expression changes that occur in cell differentiation versus tumorigenesis. After normalization and filtering of the data sets, two-way hierarchical cluster analysis identified a specific gene expression pattern associated with each cell population (Figure 3). Notably, the proportion of down-regulated genes was larger than up-regulated genes in tumor as compared to NB (61%). Conversely, the proportion of up-regulated genes was more (73%) than down-regulated genes in the differentiated layer as compared to the basal layer. These data indicate that normal differentiation and tumorigenesis take opposite paths with respect to the relative number of up- and down-regulated genes as they develop from NB cells.

**Figure 4:**
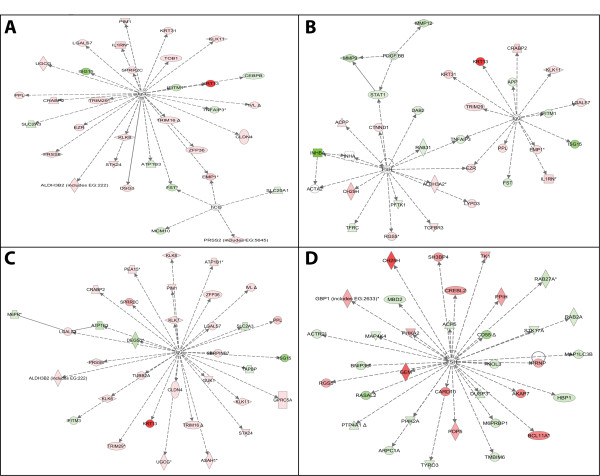
**Top network comparisons**. **A**. N vs. T - Organ Development and Nutrition Disease network. **B**. NB vs. T - Molecular Transport and Small Molecule Biochemistry network. **C**. ND vs. T - Cancer and Cellular Growth and Proliferation network. **D**. NB vs. ND - Organ Development, Nutritional Disease network. Maps were created by Ingenuity Pathway Analysis software. Red icons depict genes up-regulated; green icons depict down-regulated genes. Color intensity represents the degree of dysregulation, for example, the dark red indicates more overexpression than light red. Lines show interactions between proteins.

To further understand the expression patterns associated with each cell population, we used the computational tool Ingenuity Pathway Analysis (IPA) to investigate biological pathways to provide a more refined functional classification of genes (Additional file 1: Table S2). First, analysis of expression profiles in the cell populations in relation to pathways with known association with cancer was performed (Table 2). In general, the NB/ND comparison was involved in more pathways with a significant increase relative to other comparisons (Table 2). With respect to Diseases and Disorders of Top Biological Functions, NB/ND and T/ND were ranked in the same order with T/NB showing a unique pattern (Additional file 1: Table S2). However, instead of the Genetic Disorder pathway, Reproductive System Disease was identified as the third most related in T/NB. Although each of the comparisons was associated with cancer, they differed in the most significant sub-classification of cancer related genes: T/ND = colorectal cancer (e.g. BRAF, MET); T/NB = increased in cancer (e.g. FBXW7, PDGFA); and T/N = increased in cancer, and identified additional genes that were not present in the other comparisons (e.g. MECOM, BRCA1). Finally, biochemical pathway analysis revealed the top network for each comparison (Figure 4). Notably, the IgG network appeared on all three tumorigenesis related comparisons (T/N, T/NB, T/ND), the PDGF-BB and FSH networks were identified in the T/NB comparison, and FSH was the top network in the NB/ND comparison. In all three comparisons to T (T/N, T/NB, T/ND), ISG15 associated with the FGF Signaling pathway was the most significantly up-regulated gene in the IgG network, and was one of the top 10 up-regulated genes of the T/NB comparison (Table 1).

**Table 2:** Cancer associated pathway analysis.

Pathway	T vs. N	T vs. NB	T vs. ND	NB vs. ND
Role of BRCA1 in DNA Damage Response	6.99E-07↑	4.75E-01	3.05E-09↑	2.09E-06↑

C2M DNA Damage Checkpoint Regulation	1.03E-03↑	3.53E-01	7.46E-04↑	8.07E-06↑

p53 Signaling	1.50E-02	5.85E-02	1.77E-04↑	5.11E-06↑

G1/S Checkpoint Regulation	2.94E-02	-	4.22E-02	1.47E-03↑

PPAR signaling	6.41E-02	1.33E-01	1.78E-02	1.03E-03↓

EGF Signaling	-	1.49E-02	3.94E-01	1.49E-01

p38 MARK Signaling	1.77E-01	1.69E-01	1.74E-01	1.01E-01

PDGF Signaling	2.11E-01	7.20E-03↑	8.31E-02	7.56E-02

NF-kB Signaling	2.13E-01	6.61E-02	3.03E-01	3.35E-02

ERK/MAPK Signaling	-	3.22E-01	6.64E-02	5.20E-01

VEGF Signaling	-	-	3.15E-01	1.71E-01

IGF-1 Signaling	-	-	3.83E-01	1.28E-01

TGF-β Signaling	-	-	5.00E-01	5.09E-01

cAMP-mediated Signaling	-	-	-	4.21E-01

FGF Signaling	-	4.70E-01	-	3.62E-01

p-value comparison on cancer associated pathway

- represent no p value showed on the IPA Canonical pathways analysis.

#### DNA damage and repair

BRCA1 was up-regulated in T compared to ND (7.08 fold) and N overall (NB + ND) (3.22 fold) and up-regulated in NB compared to ND (3.7 fold); however, BRCA1 was not differentially expressed in the T/NB comparison (Table 3). This finding was corroborated by pathway analysis as the top canonical pathway in both the T/ND and NB/ND comparisons was the Role of BRCA1 in DNA Damage and Response pathway, but not in the T/NB comparison (Table 2).

**Table 3:** Role of BRAC1 in DNA Damage Repair gene expression in T vs. ND comparison

Symbol	Entrez Gene Name	Fold change
ATR	ataxia telangiectasia and Rad3 related	4.374

BACH1	BTB and CNC homology 1, basic leucine zipper transcription factor 1	-2.319

BLM	Bloom syndrome, RecQ helicase-like	2.927

BRCA1	breast cancer 1, early onset	7.08

CHEK1	CHK1 checkpoint homolog (S. pombe)	5.918

CHEK2	CHK2 checkpoint homolog (S. pombe)	2.201

E2F1	E2F transcription factor 1	2.425

E2F3	E2F transcription factor 3	5.759

E2F6	E2F transcription factor 6	2.967

FANCA	Fanconi anemia, complementation group A	2.319

FANCE	Fanconi anemia, complementation group E	2.135

FANCG	Fanconi anemia, complementation group G	2.916

HLTF	helicase-like transcription factor	6.376

MRE11A	MRE11 meiotic recombination 11 homolog A (S. cerevisiae)	2.589

MSH2	mutS homolog 2, colon cancer, nonpolyposis type 1 (E. coli)	2.049

MSH6	mutS homolog 6 (E. coli)	4.538

RAD51	RAD51 homolog (RecA homolog, E. coli) (S. cerevisiae)	4.533

RBBP8	retinoblastoma binding protein 8	2.143

RBL1	retinoblastoma-like 1 (p107)	6.574

RFC3	replication factor C (activator 1) 3, 38 kDa	4.67

RFC4	replication factor C (activator 1) 4, 37 kDa	11.105

RFC5	replication factor C (activator 1) 5, 36.5 kDa	5.366

RPA1	replication protein A1, 70 kDa	2.605

SLC19A1	solute carrier family 19 (folate transporter), member 1	2.058

STAT1	signal transducer and activator of transcription 1, 91 kDa	4.826

Similarly, other DNA damage pathways including G2M DNA Damage Checkpoint Regulation and p53 Signaling showed a similar pattern as the Role of BRCA1 in DNA Damage and Response pathway; they were up-regulated in T and NB compared to ND, with no significant difference in the T/NB comparison. These findings suggest that DNA repair pathways generally, and the BRCA1-related pathway specifically, are activated in both normal basal cells and tumor relative to differentiated cells, and might prevent or correct errors during cell division in normal cells and as a compensatory response to DNA damage in tumor cells.

### Therapeutic target identification

Due to the limited efficacy of current treatment regimens for ESCC, the identification of potential new therapeutic targets was the primary goal of the study. As a unique approach, we utilized microdissected normal basal epithelial cells (NB) as a 'normal growth' comparator for tumor (T) - in other words, a normal cell population with a relatively high growth rate. Direct comparison of these cells to rapidly growing tumor cells was performed as a biological filter to de-emphasize identification of genes associated with regular growth processes and favor identification of genes or pathways that are more closely related to tumorigenesis per se. Two pathways and twelve genes stood out as putative therapeutic targets. The PDGF signaling pathway was the most significant cancer associated pathway in the T/NB comparison (Figure 4B, Table 2), and the EGF signaling pathway was also dysregulated. Both PDGF and EGF signaling pathways have been targeted by tyrosine kinase inhibitors in some cancers, but not yet in ESCC. For example, PDGFA and PDGFRA of the PDGF signaling pathway (Table 4) are targeted by approved drugs for other cancers and potentially could be exploited for use in ESCC. Twelve differentially expressed genes were uniquely identified in the T/NB comparison (IGF2BP3↑, HLF↓, SLC6A1↑, IL1R2↓, DNASE1L↓, ASPA↓, EDN3↓, PDCD4↓, COL14A1↓, RGS5↓, DEPDC6↓, PAX5↓) with at least a 4-fold change (Additional file 1: Figure S2). In addition to genes identified in the T/NB comparison, two genes were noted to link the identified networks together. STAT1 interacts between the PDGF-BB and FSH pathways, whereas TNFAIP3 interfaces with FSH and IgG. Finally, ISG15 was specific to T in all comparisons and was one of the top 10 genes upregulated in T in compared to NB (Table 1), indicating that ISG15 is worth investigating further as a putative therapeutic target.

**Table 4:** PDGF Signaling Pathway gene expression in T vs. NB comparison

Symbol	Entrez Gene Name	Fold change
EIF2AK2	eukaryotic translation initiation factor 2-alpha kinase 2	2.099

FOS	v-fos FBJ murine osteosarcoma viral oncogene homolog	-5.056

PDGFA	platelet-derived growth factor alpha polypeptide	2.138
PDGFRA	platelet-derived growth factor receptor, alpha polypeptide	-2.62

PIK3R1	phosphoinositide-3-kinase, regulatory subunit 1 (alpha)	-2.513

PRKCA	protein kinase C, alpha	2.192

STAT1	signal transducer and activator of transcription 1, 91 kDa	2.315

### Protein expression validation

**Figure 5:**
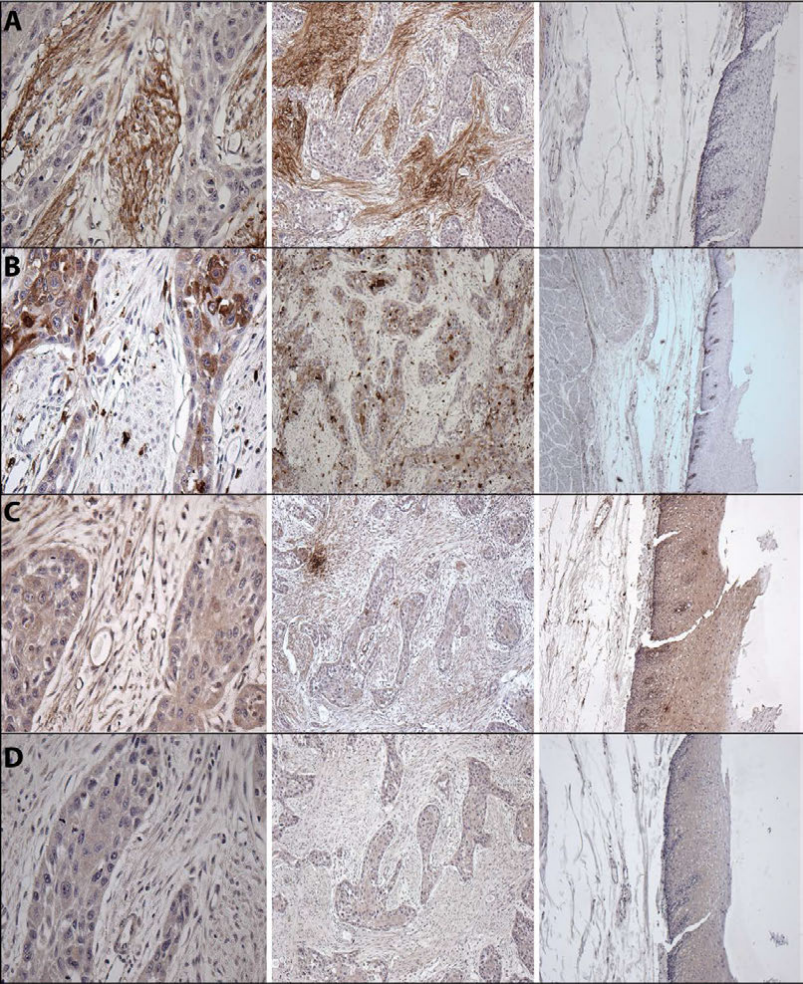
**Protein expression assessments of POSTN, ODC1, and ASPA**. Immunohistochemical staining of POSTN (**A**), ODC1 (**B**), and ASPA (**C**) are shown, with rabbit IgG (**D**) as the negative control. The left and middle panels for each represent tumor area at the magnification of 40× and 10×. The right panel for each represents the tumor adjacent normal area at a magnification of 10×

Intra-section protein expression of four selected candidate genes was compared between tumor and adjacent normal cells in 4 of the 12 ESCC cases, allowing assessment of their protein expression directly from the same slide and excluding differences attributed to the staining process. POSTN, the most overexpressed gene in the T/N comparison (Table 1), stained stroma (all 4 cases) around the tumor cells but not near normal cells. Tumor cells and normal cells were not stained (Figure 5A). Out of the 10 genes identified with available therapeutic agents, ODC1 is the only gene currently being investigated in esophageal cancer. ODC1 was positive in the cytoplasm of the tumor cells (3 from 4 cases). Tumor stroma and the normal basal layer showed weak staining (Figure 5B). ASPA was one of the twelve differentially expressed genes identified uniquely in the T/NB comparison and showed strong staining in the normal epithelium and light staining in the tumor area (3 from 4 cases) (Figure 5C). In the case of IMP3/IGF2BP3, staining was only detected in the smooth muscle cells across tumor and adjacent normal regions, but not in tumor, normal cells, or stroma (data not shown). This result did not validate the mRNA distribution pattern.

## Discussion

Laser dissection instrumentation continues to evolve and now facilitates the procurement of essentially pure populations of cells for study [19,22,29]. In parallel, the methods for analyzing small numbers of dissected cells are becoming increasingly robust and have been used successfully by many research groups, adding to the confidence that reliable data can be achieved using these methods [16,30,31]. For example, our group has developed methodologies for and demonstrated that we are able to obtain highly reproducible, statistically valid gene expression array and quantitative qRT-PCR measurements using microdissected specimens [19,21,24]. In the present study, we examined expression profiles in esophageal cancer specimens using Affymetrix expression arrays and the latest generation LCM technology to study ESCC in *ex vivo *patient samples. Because of the limited efficacy of current ESCC treatment regimes, the primary goal was to compare the expression patterns of growth-related genes in normal basal cells versus tumor cells in a search for potential therapeutic targets. As a step towards identifying therapeutic targets, we first looked at global patterns of gene expression and specific pathways associated with cell differentiation versus those associated with tumorigenesis and expression patterns of DNA damage and repair related genes. Overall, profile and pathway analysis data of cell type (T, NB, ND) comparisons revealed potential therapeutic pathway targets and individual gene targets (e.g. ISG15, IMP3, and EIF2AK2). Using the dissected T/NB cell comparison, the PDGF pathway and 12 genes unique to this comparison were identified as putative therapeutic targets for ESCC. In addition, 10 potential ESCC gene-drug pairs were identified.

### Differentiation versus tumorigenesis

To understand patterns of gene expression associated with cell differentiation versus tumorigenesis, we compared and contrasted the NB, ND, and T cell profiles. The class comparisons and PCA both revealed that ND cell samples were the most distinct based on location and distance from the basal and tumor cell populations. Moreover, the normal differentiation process (comparing NB to ND) appears to be mediated primarily by up-regulation of genes since 73% of differentially expressed transcripts were increased over basal cell levels. In contrast, the tumor cells showed primarily down-regulation (61%) of expressed transcripts compared to the NB cells, with the small percentage of up-regulated mRNAs being associated with cell growth, a pattern similar to what we have observed in other tumor types [24], and may be partially resultant from their aberrant methylation [32,33].

Regarding normal differentiation, the IPA pathway analysis showed that hypoxia signaling was dysregulated in NB/ND (p-value: 1.36E-03), without showing significant difference in the other comparisons. In response to hypoxia, activation of hypoxia-inducible factor 1 (HIF-1) occurs, and activated HIF-1 mediates cell growth, proliferation and cell death through interaction with the p53 and MDM2 pathways [34]. The NF-κB signaling pathway also uniquely differed in the NB/ND comparison. NF-κB is a protein complex that controls the transcription of DNA (Table 2) and as such regulates cell proliferation and cell survival [35]. Finally, CXCL14 was one of the top 10 up-regulated genes from the NB/ND comparison. CXCL14 is a new CXC chemokine with unknown function and receptor selectivity and is constitutively expressed in normal tissues and binds to its receptor on epithelial cells to enhance proliferation, migration, and invasion [36].

The individual gene data indicate that, as anticipated, esophageal basal cells develop into tumors through up-regulation of oncogenes and down-regulation of tumor suppressor genes. For example, transcripts for suppressors TFAP2B AP-2 *α*/β, HLF (↓7.951 fold) and EDN3 (↓5.932 fold) were decreased in tumor cells compared to basal cells (Table 1). TFAP2B is an AP-2 transcription factor that promotes normal cell apoptosis. And, a role for the AP-2 gene family in the control of cell growth and differentiation has been observed in breast cancer [37]. AP-2 *α*/β is another member of the AP-2 gene family and has also been implicated as a breast cancer suppressor gene [38]. In contrast, the oncogene IGF2BP1-3 (IMP3, ↑8.793 fold) was highly over expressed in tumor relative to NB. IMP3 normally regulates mRNA transport, translation, and turnover by binding the coding regions of target mRNAs such as IGF2 [39]. However, IMP3 expression has been correlated with increased tumor aggressiveness and reduced overall survival in pituitary tumors [40]. Our data suggest that this may also the case in ESCC and that IMP3 may be a putative therapeutic target for ESCC.

### DNA damage and repair

ESCCs exhibit extensive genomic instability, a feature that underlies their rapid growth and aggressive clinical course. As expected, we observed widespread dysregulation of genes involved in DNA repair and genomic stability in the ESCC samples with BRCA1 being one of the most over-expressed genes in tumors. Interestingly the Role of BRCA1 in DNA Damage and Response pathway was increased in both NB cells and tumor compared to ND cells. Moreover, other DNA damage pathways, including G2M DNA Damage Checkpoint Regulation and p53 Signaling, showed a similar expression pattern (Table 2).

Overall, the data indicate that important DNA repair and surveillance pathways are activated *in vivo *during both normal and pathological growth, an observation that will need to be factored into strategies that look to exploit these targets for therapeutic purposes. As a tumor suppressor gene, BRCA1 repairs damaged DNA and destroys cells when DNA cannot be repaired [41]. The G2M DNA Damage Checkpoint Regulation pathway acts to recognize damaged DNA and stop cell cycle progression at the G2/M transition, and defective G2/M checkpoints may increase cytotoxicity of chemotherapy [42]. BRCA1 is also essential in activating the Chk1 kinase that regulates DNA damage-induced G2/M transition [43]. The p53 Signaling pathway works to block cell division in response to DNA damage [44], partially through the G2/M checkpoint regulation [45]. The data suggest that DNA damage and repair pathways may not be a fruitful source of therapeutic targets.

### Therapeutic targets

We utilized a T/NB comparison to identify pathways and genes that could be putative therapeutic targets. In other words, we contrasted the expression profile of a normal dividing cell population against its counterpart transformed cell population in a search for growth-related genes that are unique to cancer and not part of the standard cell growth machinery per se, as opposed to comparing full-thickness normal epithelium that would necessarily include a high proportion of non-dividing differentiated cells [46]. As a general matter, this is an issue of concern for many studies aimed at uncovering dysregulated genes in tumors, especially common epithelial neoplasms where the majority of normal epithelium is quiescent. In ESCC however, the ability to selectively procure the self-renewing basal stem cell compartment allowed us to directly compare gene expression profiles between normally dividing cells and tumors. With regards to the concern that there is more than one cell type in the NB, we are working to adapt a new technology to esophageal specimens called expression microdissection (xMD) that facilitates procurement of molecularly-targeted sub-populations within a given tissue compartment [47,48].

Using the T/NB filter, the major cancer associated pathway in the comparison was overexpression of PDGF Signaling (Figure 4B, Table 2). Biologically, tumor growth can be promoted by PDGF via autocrine stimulation of malignant cells, by over-expression or over-activation of PDGF receptors, or by PDGF stimulation of angiogenesis within the tumor [49]. Known PDGF inhibitors such as imatinib mesylate have been used for several cancers and achieved clinical efficacy [49-51]. Our present data suggest that inhibition of the PDGF pathway may also be beneficial in treating ESCC.

In addition to potential therapeutic pathway targets, individual gene targets were also investigated. ISG15 was the most overexpressed gene in the IgG network (Figure 4) and one of the top 10 overexpressed genes in the NB/T comparison (Table 1). ISG15 is associated with the FGF Signaling pathway and involved in protein metabolism and modification and has been associated at the protein and transcriptomic levels in oral cancer [52-54] and was once associated with ESCC [55], suggesting that inhibition of ISG15 may be beneficial in treatment. Overall, 12 genes were identified as being specific to the T/NB comparison and represent unique therapeutic targets. As evidenced by the clinical success of imatinib, gefitinib, and erlotinib, kinase family gene targets may be a useful place to begin investigating new therapeutic agents. The data identified several kinases that are up-regulated in tumor cells, including; MET, PFTK1, BUB1, CKS1B, EIF2AK2, and NEK2. Interestingly, EIF2AK2 regulates TP53; TP53 regulates BUB1 and NEK2; and NEK2 regulates BRCA1. Therefore, potentially targeting upstream at EIF2AK2 may have a good chance of therapeutic efficacy in ESCC.

We also identified the following sets of gene-drug pairs, i.e., therapeutic agents that act as appropriate inhibitors or agonists with identified tumor dysregulated genes: 1) AURKA, targeted by MLN8054, inhibitor; 2) CDC2, targeted by flavopiridol, inhibitor; 3) PDGFRA, targeted by becaplermin, agonist; 4) PDGFA, targeted by cilostazol, inhibitor; 5) IL8RB, targeted by SB-265610, agonist; 6) NPR3, targeted by nesiritide, agonist; 7) NR3C2, targeted by fludrocortisones acetate, agonist; 8) ODC1, targeted by eflornithine, inhibitor; 9) PRKCA, targeted by L-threo-safingol, inhibitor; and 10) SOAT1, targeted by pactimibe, inhibitor. Of these 10 genes and therapeutic agent combinations, only one is currently being investigated in esophageal cancer: ODC1/eflornithine, of which a phase II clinical trial (NCT00003076) was recently completed although study results have not been posted yet. However, targeting of ODC1 has been successfully used in a combination therapy trial where a low dose of chemopreventive drugs difluoromethylornithine (DFMO; targeting ODC1) plus sulindac has shown notable efficacy in preventing colorectal adenoma recurrence [56] with few side effects.

From the above potential therapeutic targets, four genes were selected for further protein level assessment by IHC. ODC1 showed a consistent distribution pattern at both the mRNA and protein level. ASPA exhibited strong staining in the normal epithelium compared to tumor as expected, but did not show increased staining in the NB compared to ND. The 2-fold mRNA overexpression of ASPA may be insufficient to be reflected at the protein level. Since POSTN was reported as an extracellular protein [57], the staining of the stroma around the tumor implied that the tumor cells secrete POSTN to tumor stroma. This observation is consistent with the array data as tumors expressed over 10-fold more POSTN than normal esophageal epithelium. Similar immunostaining of POSTN was also reported by Kwon et al. [58] and there are recent reports of the role of POSTN in pancreatic, ovarian and gastric cancer, and potential agents are noted to influence its expression [59]. Overall, the mRNA expression pattern of three of the four selected genes was validated at the protein level.

## Conclusions

The current study used a unique tissue microdissection strategy and microarrays to measure gene expression profiles associated with cell differentiation versus tumorigenesis in twelve cases of patient-matched normal basal squamous epithelial cells (NB), normal differentiated squamous epithelium (ND), and squamous cell cancer. We observed that ND expression patterns were markedly different from NB and tumor; whereas, tumor and NB were more closely related. Tumors showed a general decrease in differentially expressed genes relative to NB as opposed to ND that exhibited the opposite trend. In addition, the FSH and IgG networks were most highly dysregulated in normal differentiation and tumorigenesis. DNA repair pathways were generally elevated in NB and tumor relative to ND indicating involvement in both normal and pathological growth. Moreover, PDGF signaling pathway and 12 individual genes unique to the tumor/NB comparison were identified as therapeutic targets. We further examined the protein expression level and the distribution patterns of four genes: ODC1, POSTN, ASPA and IGF2BP3, and three (ODC1, POSTN, ASPA) were altered at the protein level. The analysis of microdissected normal and pathological cell populations from *ex vivo *clinical samples has revealed new insights into the genes and molecular pathways that mediate ESCC development, demonstrated the feasibility of using a microdissection-based strategy to identify novel therapeutic targets, and may be an effective strategy for studying various cancer types more generally. Certainly however, further validation of the identified genes and pathways is needed to confirm the present findings in a larger cohort of patients before conclusions can be drawn about therapeutic targets, and the issue of tumor heterogeneity will also need to be explored for candidate differentially expressed genes.

## Abbreviations

ESCC: Esophageal squamous cell carcinoma; NB: Normal basal squamous epithelial cells; ND: Normal differentiated squamous epithelium; T: Tumor; LCM: Laser capture microdissection; IPA: Ingenuity pathway analysis; PCA: Principal components analysis; IHC: Immunohistochemistry.

## Competing interests

The authors declare that they have no competing interests.

## Authors' contributions

WY, MRE-B and HSE Conceived and designed the experiments. WY, JRC, MAT, KY and AP performed the experiments. WY, JHS, JH, MRE-B and HSE analyzed the data. WY, JHS, NH, AMG, PRT, MRE-B and HSE contributed reagents/materials/analysis tools. WY, MRE-B and HSE wrote and revised the manuscript. All authors read and approved the final manuscript.

## Supplementary Material

Additional file 1 **Table S1**. Sample microdissection and RNA assessment. **Table S2**. Pathway analysis summary. **Table S3**. Clinical annotation of the twelve ESCC cases studied. **Figure S1**. Quality control assessment of each array using normalized unscaled standard error (NUSE) and relative log expression (RLE). × axis represents individual cases; Y axis represents NUSE median and RLE median separately. **Figure S2**. Venn diagram across NB/T, ND/T and N/T. Input data are those differential expressed genes from each comparison with ≥ 4-fold change. Click here for file
