# Supplementary material for: Identification of unique expression signatures and therapeutic targets in esophageal squamous cell carcinoma
Source: BMC Res Notes. 2012 Jan 26;5:73. doi: 10.1186/1756-0500-5-73 (PMC3283499; doi:10.1186/1756-0500-5-73)
Supplement: Additional file 1 — Table S1. Sample microdissection and RNA assessment. Table S2. Pathway analysis summary. Table S3. Clinical annotation of the twelve ESCC cases studied. Figure S1. Quality control assessment of each array using normalized unscaled standard error (NUSE) and relative log expression (RLE). × axis represents individual cases; Y axis represents NUSE median and RLE median separately. Figure S2. Venn diagram across NB/T, ND/T and N/T. Input data are those differential expressed genes from each comparison with ≥ 4-fold change. [file 1756-0500-5-73-S1.DOC]

**Additional file 1:**

**Table S1. Sample microdissection and RNA assessment.**

|  |  |  | **Total RNA (≈18ul)** | | **Round 2 aRNA** |
| --- | --- | --- | --- | --- | --- |
| **Case** | **Sample** | **LCM shots** | **ng/ul** | **RIN** | **(≈100ul) ug/ul** |
| 1 | N-B | 4000 | 3.47 | 6.3 | 1.293 |
|  | N-D | 4000 | 7.88 | 5.9 | 0.232 |
|  | T | 3200 | 3.27 | 7.4 | 1.093 |
| 2 | N-B | 4600 | 11.76 | 6.4 | 0.997 |
|  | N-D | 4000 | 3.43 | 6.7 | 1.1 |
|  | T | 4800 | 7.64 | 6.4 | 0.515 |
| 3 | N-B | 5200 | 14.72 | 5.9 | 0.995 |
|  | N-D | 5000 | 18.7 | 6.6 | 0.417 |
|  | T | 5000 | 5.31 | 6.8 | 1.069 |
| 4 | N-B | 8000 | 3.21 | 5.9 | 0.956 |
|  | N-D | 8000 | 1.75 | 7.4 | 1.053 |
|  | T | 4000 | 5.05 | 5.6 | 0.523 |
| 5 | N-B | 4000 | 3.36 | 5.5 | 1.309 |
|  | N-D | 8000 | 1.65 | 8.7 | 1.197 |
|  | T | 8000 | 2.8 | 7.7 | 1.226 |
| 6 | N-B | 8000 | 2.73 | 5 | 0.452 |
|  | N-D* | 4000 | 4.49 | 4.6 | 1.427 |
|  | T | 4000 | 4.12 | 5.3 | 1.273 |
| 7 | N-B | 4000 | 8.23 | 7.2 | 1.111 |
|  | N-D | 8000 | 1.75 | 8.7 | 1.213 |
|  | T | 4000 | 5.39 | 5.3 | 1.033 |
| 8 | N-B | 4000 | 3.88 | 6.5 | 1.279 |
|  | N-D | 4000 | 9.21 | 8.1 | 0.671 |
|  | T | 8000 | 3.1 | 8 | 1.203 |
| 9 | N-B | 4000 | 3.46 | 7.4 | 1.411 |
|  | N-D | 8000 | 2.08 | 8.7 | 1.321 |
|  | T | 4000 | 14.82 | 7.8 | 1.148 |
| 10 | N-B | 4000 | 5.6 | 6.3 | 1.238 |
|  | N-D | 4000 | 4.24 | 6.4 | 1.385 |
|  | T | 8000 | 2.41 | 6.8 | 1.291 |
| 11 | N-B | 4000 | 4.2 | 4.2 | 0.458 |
|  | N-D | 8000 | 1.55 | 6.6 | 1.174 |
|  | T | 4000 | 3.35 | 5.2 | 1.316 |
| 12 | N-B | 4000 | 17.08 | 5.7 | 0.674 |
|  | N-D | 4000 | 5.34 | 6.2 | 0.944 |
|  | T | 4000 | 5.05 | 7.4 | 0.894 |
| Note: Laser Capture Microdissection with midsize laser spot. | | | | | |
| * This sample did not pass array quality control criteria. | | | | |  |

**Table S2.**  **Pathway analysis summary.**

| **Comparison** | **Network and Functions** | **p-value** | **# of genes** | **Score*** |
| --- | --- | --- | --- | --- |
| **T/N**** | **A. Top networks** |  |  |  |
|  | Hair and Skin Development and Function, Organ |  |  |  |
|  | Development, Nutritional Disease |  |  | 29 |
|  | Organismal Injury and Abnormalities, Cell-To- cell |  |  |  |
|  | Signaling and Interaction, Cell-mediated Immune Response |  |  | 23 |
|  | Cellular Movement, Immune Cell Trafficking |  |  | 17 |
|  | **B. Top Biological Functions** |  |  |  |
|  | 1). Disease and Disorders |  |  |  |
|  | Cancer | 1.07E-30 - 7.10E-03 | 423 |  |
|  | Gastrointestinal Disease | 2.66E-27 - 6.09E-03 | 182 |  |
|  | Genetic Disorder | 1.11E-22 - 5.46E-03 | 609 |  |
|  | 2). Molecular and Cellular Function |  |  |  |
|  | Cell Cycle | 1.62E-28 - 7.10E-03 | 146 |  |
|  | Cell Death | 3.33E-16 - 5.05E-03 | 196 |  |
|  | Cellular Growth and Proliferation | 4.56E-12 - 6.22E-03 | 235 |  |
|  | 3). Physiological System Development and Function |  |  |  |
|  | Tissue Development | 1.50E-09 - 5.79E-03 | 80 |  |
|  | Hair and Skin Development and Function | 1.73E-06 - 4.68E-03 | 28 |  |
|  | Organ Development | 1.73E-06 - 4.68E-03 | 18 |  |
|  | **C. Top Canonical Pathways** |  |  |  |
|  | Fatty Acid Metabolism | 2.82E-07 |  |  |
|  | Role of BRCA1 in DNA Damage Response | 6.99E-07 |  |  |
|  | Role of CHK Proteins in Cell Cycle Checkpoint Control | 6.92E-06 |  |  |
| **T/NB** | **A. Top networks** |  |  |  |
|  | Lipid Metabolosm, Molecular Transport, Small Molecule |  |  |  |
|  | Biochemistry |  |  | 33 |
|  | Cellular Movement, Immune Cell Trafficking, Cellular |  |  |  |
|  | Growth and Proliferation |  |  | 27 |
|  | Inflammatory Disease, Organismal Injury and |  |  |  |
|  | Abnormalities, Skeletal and Muscular Disorders |  |  | 15 |
|  | **B. Top Biological Functions** |  |  |  |
|  | 1). Disease and Disorders |  |  |  |
|  | Cancer | 3.72E-21 - 2.77E-02 | 201 |  |
|  | Gastrointestinal Disease | 9.15E-12 - 2.48E-02 | 85 |  |
|  | Reproductive System Disease | 1.89E-09 - 2.51E-02 | 149 |  |
|  | 2). Molecular and Cellular Function |  |  |  |
|  | Cell Cycle | 2.43E-12 - 2.88E-02 | 61 |  |
|  | Cell Death | 2.69E-12 - 2.88E-02 | 97 |  |
|  | Cellular Growth and Proliferation | 3.08E-08 - 2.83E-02 | 115 |  |
|  | 3). Physiological System Development and Function |  |  |  |
|  | Skeletal and Muscular System Development and Function | 7.10E-06 - 2.70E-02 | 19 |  |
|  | Hair and Skin Development and Function | 1.72E-05 - 2.48E-02 | 27 |  |
|  | Organ Development | 1.72E-05 - 1.39E-02 | 15 |  |
|  | **C. Top Canonical Pathways** |  |  |  |
|  | Fatty Acid Metabolism | 1.26E-07 |  |  |
|  | Tryptophan Metabolism | 1.91E-07 |  |  |
|  | Arachidonic Acid Metabolism | 3.16E-06 |  |  |
| **T/ND** | **A. Top networks** |  |  |  |
|  | Cancer, Cellular Growth and Proliferation, Neurological Disease |  |  | 22 |
|  | Cell Morphology, Cellular Assembly and Organization, |  |  |  |
|  | Cellular Function and Maintenance |  |  | 22 |
|  | Organismal Injury and Abnormalities, Antigen |  |  |  |
|  | Presentation, Cellular Movement |  |  | 15 |
|  | **B. Top Biological Functions** |  |  |  |
|  | 1). Disease and Disorders |  |  |  |
|  | Cancer | 6.38E-29 - 1.12E-02 | 840 |  |
|  | Gastrointestinal Disease | 6.38E-29 - 1.09E-02 | 283 |  |
|  | Genetic Disorder | 7.29E-26 - 8.56E-03 | 326 |  |
|  | 2). Molecular and Cellular Function |  |  |  |
|  | Cell Death | 1.75E-22 - 1.11E-02 | 374 |  |
|  | Cell Cycle | 2.63E-22 - 1.12E-02 | 221 |  |
|  | Cellular Growth and Proliferation | 5.32E-12 - 1.09E-02 | 435 |  |
|  | 3). Physiological System Development and Function |  |  |  |
|  | Tissue Development | 9.47E-07 - 4.62E-03 | 120 |  |
|  | Hair and Skin Development and Function | 4.55E-06 - 4.85E-03 | 50 |  |
|  | Organ Development | 5.99E-05 - 5.99E-05 | 21 |  |
|  | **C. Top Canonical Pathways** |  |  |  |
|  | Role of BRCA1 in DNA Damage Response | 3.05E-09 |  |  |
|  | Pyrimidine Metabolism | 3.29E-07 |  |  |
|  | Role of CHK Proteins in Cell Cycle Checkpoint Control | 3.66E-06 |  |  |
| **NB/ND** | **A. Top networks** |  |  |  |
|  | Hair and Skin Development and Function, Organ |  |  |  |
|  | Development, Nutritional Disease |  |  | 28 |
|  | Cell Signaling, Post-Translational Modification, Small |  |  |  |
|  | Molecular Biochemistry |  |  | 28 |
|  | Cancer, Cell Death, Connective Tissue Disease |  |  | 13 |
|  | **B. Top Bio Functions** |  |  |  |
|  | 1). Disease and Disorders |  |  |  |
|  | Cancer | 3.94E-24 - 1.12E-02 | 533 |  |
|  | Gastrointestinal Disease | 1.17E-19 - 6.58E-03 | 181 |  |
|  | Genetic Disorder | 1.38E-18 - 1.11E-02 | 235 |  |
|  | 2). Molecular and Cellular Function |  |  |  |
|  | Cell Cycle | 1.74E-15 - 1.12E-02 | 144 |  |
|  | Cell Death | 6.04E-12 - 1.12E-02 | 227 |  |
|  | Cellular Growth and Proliferation | 6.99E-10 - 1.02E-02 | 120 |  |
|  | 3). Physiological System Development and Function |  |  |  |
|  | Hair and Skin Development and Function | 7.98E-06 - 7.79E-03 |  |  |
|  | Organ Development | 7.98E-06 - 7.98E-06 |  |  |
|  | Connective Tissue Development and Function | 1.49E-05 - 9.24E-03 |  |  |
|  | **C. Top Canonical Pathways** |  |  |  |
|  | Role of BRCA1 in DNA Damage Response | 2.09E-06 |  |  |
|  | p53 Signaling | 5.11E-06 |  |  |
|  | Cell Cycle: G2M DNA Damage Checkpoint Regulation | 8.07E-06 |  |  |

* Score = numerical value used to rank networks according to degree of relevance to the Network Eligible Molecules in the data set (defined by IPA). ** N = NB + ND

| **Table S3. Clinical annotation of the twelve ESCC cases studied** | | | | | | | |  |  |
| --- | --- | --- | --- | --- | --- | --- | --- | --- | --- |
| **Cases** | **Age/sex** | **Tumor** | **Stage/** | **#positive/** | **FH of any** | **Smoking** | **Alcohol** | **Pickled** | **Survival** |
|  |  | **location** | **grade** | **#examine** | **cancer** |  | **drinking** | **vegetable** | **months** |
| 1 | 62/F | Middle | 3/2 | 0/4 | N | Y | Never | < Monthly | 39.0 |
| 2 | 63/M | Middle | 3/2 | 0/19 | N | Y | Daily | Never | 23.3 |
| 3 | 52/M | Middle | 2/1 | 4/16 | Y | Y | Weekly | Daily | 58.1 |
| 4 | 63/M | Middle | 3/1 | 0/11 | N | N | < Weekly | Daily | 23.1 |
| 5 | 55/F | Middle | 2/2 | 17/21 | N | N | Never | < Monthly | 5.9 |
| 6 | 52/M | Middle | 3/2 | 0/10 | Y | Y | < Weekly | Daily | 42.5 |
| 7 | 64/M | Middle | 3/1 | 1/21 | N | Y | Daily | Weekly | 41.0 |
| 8 | 48/F | Middle | 3/3 | 2/7 | N | N | < Weekly | Never | 86.1 |
| 9 | 49/F | Middle | 2/2 | 1/5 | Y | N | Never | < Monthly | 29.6 |
| 10 | 61/M | Middle | 2/2 | 1/18 | Y | N | Weekly | < Monthly | 19.8 |
| 11 | 68/M | Middle | 3/2 | 7/11 | N | Y | < Weekly | Never | 16.2 |
| 12 | 50/F | Middle | 3/3 | NR/NR | N | N | Never | Never | 5.7 |
| Note: NR = missing data | | |  |  |  |  |  |  |  |

**Supplemental Figure Legends**

**Figure S1.** Quality control assessment of each array using normalized unscaled standard error (NUSE) and relative log expression (RLE). X axis represents individual cases; Y axis represents NUSE median and RLE median separately.

**Figure S2.** Venn diagram across NB/T, ND/T and N/T. Input data are those differential expressed genes from each comparison with > 4-fold change.

Yan, et al.

Figure S1.

**Cases**

**Median**

Yan, et al.

Figure S2
